# Heavy Metals Content in Selected Medicinal Plants Produced and Consumed in Serbia and Their Daily Intake in Herbal Infusions

**DOI:** 10.3390/toxics11020198

**Published:** 2023-02-20

**Authors:** Irina Kandić, Milan Kragović, Jelena Petrović, Peđa Janaćković, Milan Gavrilović, Miloš Momčilović, Marija Stojmenović

**Affiliations:** 1 “Vinča” Institute of Nuclear Sciences, National Institute of the Republic of Serbia, University of Belgrade, 11351 Belgrade, Serbia; 2 Faculty of Biology Chair of Morphology and Systematics of Plants, University of Belgrade, Studentski trg 16, 11000 Belgrade, Serbia

**Keywords:** medicinal herbs, heavy metals, ICP-OES, daily intake, health risk, human health

## Abstract

The heavy metals content (HMs) was investigated in 14 different medicinal plants collected from the three regions in Central Serbia, Zlatar, Sokobanja, and Kopaonik. The concentrations of Cd, Cr, Ni, Hg and Pb were determined: Cd (<0.03–2.72 mg/kg); Cr (<0.08–12.1 mg/kg); Ni (<0.08–12.2 mg/kg); Pb (0.6–49.0 mg/kg); the Hg concentration was below the detection limit of 0.06 mg/kg in all samples. The daily intake of HMs due to ingestion of 200 mL of herbal infusion was in all cases below the recommended limit prescribed by the World Health Organization. The estimated daily intake values were below the values for the oral reference dose regulated by the U.S. Environmental Protection Agency (USA EPA). The target hazard quotient and hazard index for Cd, Cr Ni, and Pb were below 1. Nevertheless, due to the tendency of heavy metals to accumulate in the organism, attention should be paid to the daily intake of herbal infusion during long-term usage. Specifically, it is recommended to consume not more than one cup (200 mL) of infusion per day made from thyme (Mt. Zlatar) and blueberry (Mt. Kopaonik), and not more than two cups per day for other herbs.

## 1. Introduction

In recent years herbal medicine has been considered a dietary supplement for disease prevention and as an alternative/complementary medicine. In addition, the utilization of many herbal preparations has highly increased due to their fewer side effects compared to synthetic drugs. Medicinal plants have a significant role in the everyday life of rural people, particularly in developing countries. In many Balkan countries, people still practice herbal traditional medicine. Serbia is located in the north-central region of the Balkan Peninsula and has a flora of 4 246 taxa [1] of which 1000 to 1500 are used as medicinal plants, food stuffs, spices, food preservatives, natural dyes or additives [2]. It should be noted that geography and environmental conditions have important impacts on the medicinal efficacy of plants, including the content of trace elements in them.

Trace elements are important for the formation of active chemical constituents of medicinal plants and therefore influence both their healing properties and toxicity [3]. Very small quantities of essential trace elements are necessary for a human to perform their vital metabolic activities [4,5]. Although some trace elements are essential in low concentrations for biological systems [6], higher concentrations of some of them are toxic to hu-mans. Moreover, some of them, similar to heavy metals (Ag, As, Cd, Cr, Hg, Ni, Pb, and Sn) are toxic, or even lethal, in low concentrations. According to the European Food Safety Authority (EFSA), the tolerable weekly intake (TWI) of Cd was 2.5 µg per kg of body weight [7], while the value for Pb is estimated at 0.68 µg per kg of body weight per day, which is based on middle bound mean Pb occurrence [8]. In addition, according to EFSA, the TWI for inorganic Hg was 4 µg per kg of body weight expressed as mercury [9]. In regard to chromium, the maximal proposed use levels were 2 g/day for children from 3 to 9 years old and 4 g/day thereafter [10], while for nickel, the lowest-observed-adverse-effect-level of 4.3 μg Ni/kg body weight was selected as the reference point [11].

It is well known that most medicinal plants should be used for an extended period of time to produce positive significant effects on human health, although in this way the harmful heavy metals (HMs), potentially present in them, can accumulate in the human body, and induce numerous health problems, e.g., weakening of the immune system, cardiac dysfunction, fetal malformation, impaired psychological and neurological behavior, and gastrointestinal cancer [12]. Many heavy metals and their compounds, such as Cd, Cd compounds and Ni compounds (Group I carcinogens), Pb compounds (inorganic) (Group 2A carcinogens), and Ni (metallic) and Pb (Group 2B carcinogens), have been classified as carcinogens by the International Agency for Research on Cancer (IARC) [13]. Methylmercury compounds according to the IARC classification belong to Group 2B carcinogens, while Hg and inorganic Hg compounds were not classifiable as to their carcinogenicity to humans. In high concentrations, Cd negatively affects the kidney, liver, vascular, and immune systems. Small concentrations of Cr and Ni are essential for human metabolism. These elements are moderately labile in soil and produce a toxic effect on human health only if consumed plants are grown on contaminated soil [6]. In larger concentrations, and (or) after prolonged exposure, Cr and Ni can produce carcinogenic, mutagenic, embryotoxic, and teratogenic effects on human health [14]. Pb poisoning is known to be one of the global environmental and public health hazards. Pb can cause many adverse effects on the blood, nervous, immune, renal, skeletal, muscular, reproductive, and cardiovascular systems [12]. Hg also has pronounced toxic effects, including teratogenic, carcinogenic, and mutagenic ones.

Although herbal medicine is officially recognized in many countries as beneficial to human health, the guidelines and regulations for its safety usage are scarce. Unlike pharmaceuticals, there are few scientific studies dealing with the safety conditions of herbal products consumption. The HMs in medicinal plants have been highly investigated in recent years [15,16,17,18]. Ražić and Kuntić [19] investigated nine elements including Cd, Hg, and Pb in herbal infusions, which are widely consumed for medical purposes in Serbia, and concluded that the contents of toxic elements (As, Cd, and Pb) in almost all samples were below the maximum permissible levels. Mihaljev et al. [20], also investigated the content of Ni, and some other elements in medicinal herbs in Serbia and found that investigated samples of herbal infusions were considered safe for human consumption.

The precise knowledge of HM content in herbal infusion is essential for the estimation of whether the ingested heavy metal concentration will be within the prescribed values, or not, and how it will affect the health of consumers [21]. There are many papers dealing with HM content in herbs, but few of them report the HM’s daily dose ingested in herbal infusions to determine safe daily intake for consumers.

In our previous study, we investigated in detail the activity concentration of ^137^Cs, ^40^K, and ^210^Pb radionuclides as potentially dangerous pollutants for human health, in selected medicinal plants from the same three regions in Central Serbia [22,23]. The values of individual annual effective doses due to ingestion of 200 mL of infusion are less than 100 μSv for each type of medicinal plant. This indicates that daily use of herbal infusion in the amount of 200 mL for one year does not represent a radiological risk for health. However, an individual annual effective dose of ^210^Pb in herbal infusion in the amount of 200 mL is for some herbs between 21.6 μSv (blueberry; Sokobanja) and 38.3 μSv (thyme; Mt. Zlatar) [22]. This indicates that consumption of larger amounts of infusion daily (2–3 cups of 200 mL) over one year could exceed the recommended dose level.

In order to complement the research regarding the potential health risk of well-known medicinal plants from the Republic of Serbia, we expanded our research to examine the presence of HMs in selected medicinal plants and their impact on human health. We analyzed the content of the three most toxic elements: Cd, Hg, and Pb, as well as Ni and Cr, which in small amounts are beneficial to human health. Therefore, the aim of this study was: (1) to determine the content of HMs in selected medicinal plants commonly used in traditional medicine from the three mentioned regions (Mt. Kopaonik, Sokobanja, and Mt. Zlatar) in Central Serbia, and (2) to calculate the daily intake of HMs in herbal infusions made from these herbs, and (3) to assess the potential health risks of the consumption of these herbal infusions based on the estimated daily intake (EDI), target hazard quotient (THQ), and hazard index (HI) and (4) to raise awareness in people that potentially higher concentrations of HMs in medicinal herbs and their infusions can have a negative impact on human health with long term consumption.

## 2. Materials and Methods

### 2.1. Preparation of Herbal Samples for Determination of Heavy Metals

The fourteen selected wild medicinal herbs (Table 1) were procured in dried form at local markets in the Republic of Serbia. The investigated medicinal plants were collected and produced from three locations in Central Serbia (regions of Mt. Zlatar, Sokobanja, and Mt. Kopaonik). All investigated medical plants (14 selected herbs from three locations, a total of 42 samples) contained a manufacturer’s specification following the Rulebook on the quality of tea, herbal tea, and their products of the Republic of Serbia and the Law regulating food safety of the Republic of Serbia [24,25]. The manufacturer’s specification contained the registration number, date of adoption, product and trade name, a brief description of the manufacturing process, of preparation of tea basic quality requirements, a report on health and quality testing (physical, chemical, and sensory properties), month and year of collecting, the production date, as well as data from the declaration, following the law governing food safety. The herbal tea declaration also contained the Latin name of the plant. Additionally, the tested herbs were also identified at the Department of Morphology and Systematics of Plants of the Faculty of Biology, University of Belgrade. The herbal samples were homogenized in a laboratory blender and sieved using a mesh sieve.

### 2.2. The Method and Standards for Determination of Heavy Metals

To determine heavy metal amounts, 250 mg of each sample was taken and put in a closed PFA digestion vessel and then 7 mL of 70% nitric acid (Macron Fine Chemicals) and 1 mL of 30% hydrogen-peroxide (Sigma-Aldrich, St. Louis, MO, USA) were added. The samples were then digested in the Jupiter-A (Sineo Microwave Chemistry Technology, China) microwave oven according to the following procedure: in 10 min the temperature raised up to 150 °C, and after that in 20 min up to 190 °C, where it was held for 10 min. Each sample was prepared in duplicate, and a blank was prepared in order to discriminate possible impurities. After digestion, the vessels were cooled down at room temperature, transferred to volumetric flasks, and diluted to 20 mL with deionized water. All solutions were stored in polyethylene flasks till the measurements of HMs by inductively coupled plasma optical emission spectrometry (ICP-OES) on the Thermo Scientific™ iCAP™ 7400 ICP-OES analyzer.

The ICP-OES method was applied as a technique of choice for the determination of diverse trace elements in plants as a standard and leading spectrochemical technique. The single element mercury (Hg) 1000 ppm calibration standard obtained from J.T. Baker and the Multi-element ICP IV 1000 ppm standard obtained from AccuStandard were used to prepare the set of calibration standards for the ICP-OES analysis. All measurements were repeated three times. The limits of detection (LOD) were (in mg/kg): <0.03 for Cd, <0.08 for Cr, <0.08 for Ni, <0.7 for Pb, and <0.06 for Hg.

### 2.3. The Risk Assessment of Toxic Metals Intake through Herbal Infusion

#### 2.3.1. Daily Intakes (D) of Heavy Metals by Consuming 200 mL Herbal Infusion

The daily intake D of a metal [µg] was calculated according to the formula:

*D* = *C* × *C_d_* × *m* (1)

where *C* is the metal content in the herb (mg/kg), *C_d_* is the metal solvation coefficient during the infusion preparation (Table S1; [26]), m is the amount of herb used for preparation of the 200 mL herbal infusion, which is usually about 0.002 kg. The value of *C_d_*, the metal solvation coefficient during the infusion preparation for Hg was not determined in investigations conducted by Ababneh et al. [26].

#### 2.3.2. The Estimated Daily Intake (EDI) of Heavy Metals Based on the Ingestion Rate of Herbal Infusion

The health risk of herbal infusion as a result of exposure to Cd, Cr, Ni, Hg, and Pb through herbal infusion intake was also evaluated based on the estimated daily intake (*EDI*), target hazard quotient (*THQ*), and hazard index (*HI*) [6,27,28]. The calculation of estimated daily intake was based on the following equation:

(2) $$EDI_{i}=\frac{\left(C_{i}\times IR\times TR_{i}\right)}{\left(BW\times 100\right)}$$

In this equation, *C_i_* is the metal concentration in the medicinal herb (mg/kg), *i* is the toxic metal type (1, 2, 3, 4, 5), and *IR* is the ingestion rate of herbal infusion for adults (11.4 g/person/day) [6,27,29], *TR_i_* is the transfer rate of toxic metal from the medicinal herb into the infusion [6], BW is body weight (60 kg for adults) [6,30]. The transfer rate of toxic metal from the medicinal herb into the infusion is presented in Table S2.

#### 2.3.3. Health Risk of Individual Toxic Metals through Intake of Herbal Infusion

The target hazard quotient (*THQ*) represents the quantitative evaluation of the potential non-carcinogenic effects of individual toxic metals [6,28] and was calculated according to equation:

(3) $$THQ_{i}=\frac{EDI_{i}}{RfD_{i}}$$

where *EDIi* is the daily average exposure dose (mg/kg/day) and *RfDi* (mg/kg/day) represents the oral reference dose regulated by the U.S. Environmental Protection Agency (USA EPA), presented in Table S3.

#### 2.3.4. The Total Risk of Multiple Toxic Metals through Intake of Herbal Infusion

The hazard index (*HI*) was used to estimate the total impact of toxic metals from the herbal infusion. The hazard impact represents the total health risk related to the toxic metals and it was estimated according to the equation:

(4) $$HI=THQ_{1}+THQ_{2}+THQ_{3}+THQ_{4}+THQ_{5}$$

*THQi* represents the *THQ* value of an individual toxic metal. If the value of *HI* is less than 1 the exposure dose is lower than the adverse reaction threshold, and if the *HI* is higher than 1 it is possible that toxic metals will have negative effects on human health [6,28].

## 3. Results and Discussion

### 3.1. ICP-OES Analysis

The concentrations of Cd, Cr, Ni, Hg, and Pb in 14 selected medicinal herbs from the three regions of Central Serbia (Mt. Zlatar, Sokobanja, and Mt. Kopaonik) are presented in Table 2. To get better insight into HM concentrations in medicinal herbs, except for results of concentrations of HM in selected medicinal herbs from the Republic of Serbia [28,31], herein we compared our results with results from some of the corresponding herbs from Turkey (Kayseri) [32], as one of the areas in which teas from the investigated plants are often consumed. The results showed that some significant differences exist between the various herbs in HM concentration, depending on whether the plant is annual, or perennial (all selected medicinal herbs investigated in our paper are perennial), the used part, and plant families. The perennial herbs, such as thyme, blueberry, dog rose, or winter savory, which accumulate HMs predominantly in roots, and subsequently transfer some portions to other parts of the plant [33,34], can be expected to have higher concentrations of HMs. However, according to our analysis, the HM concentrations in these herbs highly depend on the location and the type of HM. Generally, according to the literature, the HM accumulation in various parts of plants usually follows the patterns: root > leaf > shoot (stem) > fruit; and lateral root > main root, old leaf > young leaf [35]. The difference in the HM concentrations also exist between the same herbs from different locations, suggesting that soil in the areas from where the investigated herbs were collected is quite different in some aspect. The overall location (G) trend of the investigated HM concentrations per herb is mainly: Zlatar > Kopaonik > Sokobanja.

Heavy metals are commonly persistent in nature, and therefore accumulate in soils and herbs [36]. Besides natural origins, HMs may enter the soil from different artificial sources, such as mineral fertilizers, industry, urban areas, traffic, etc. Thus, the content of HMs in plants can be a consequence of many factors: soil type and texture, details of precipitation, pH of the environment, chemical form in which the elements are present in soil and plants, and plant origin. Acidic soils especially, increase the mobility of HMs, and their uptake by herbs, particularly of Pb [37]. Therefore, the concentration level of HMs accumulated in investigated herbs can be an indication of soil quality, but also of human exposure to them. Accordingly, the World Health Organization (WHO, 2007) [38] established national limits for heavy metals in plants (Table 3).

#### 3.1.1. Cadmium and Chromium

In numerous examined herbs from all three locations, the Cd content was generally low, and below the LOD of <0.03 mg/kg (Table 2). The highest Cd concentration of 2.72 mg/kg was found in thyme from Zlatar, while the Cd concentration in thyme from Kopaonik and Sokobanja was below the LOD. Kopaonik had the lowest average Cd concentration per herb, while Zlatar had the highest. Ražić and Kuntić [19] also found that the content of Cd in various medicinal herbs was below the maximum permissible level, except for peppermint (8.61 mg/kg). de Oliveira et al. [30] also found a low Cd content (below 0.19 mg/kg) per herb. In the research conducted by Popović et. al. [31] the highest concentrations of Cd were in St. John’s wort from the Republic of Serbia (0.93 μg/g). Similar concentration values of Cd were also recorded by Krstić et al. [28] (0.927 mg/kg). In our study, the results for the Cd contents in St. John’s wort samples from all three locations were: 2.00 mg/g, 0.35 mg/g, and 0.83 mg/g for Zlatar, Sokobanja, and Kopaonik, respectively. Thus, the sample from Zlatar showed the highest content of Cd and significantly higher values compared to the results of Krstić et al. [28] and Popović et. al. [31]. Namely, St. John’s wort is characterized as a Cd accumulator [39,40]. Karak and Bhagat [41] reported that the presence of phosphate fertilizer may be a source of Cd in soil, while the presence of increased lead in soil stimulates their absorption by plants. Generally, many factors can influence Cd absorption by plants. However, plants that contain an increased concentration of Cd are not always hazards for health, but that depends on the efficiency of extraction of Cd in the infusion [42]. Although certain types of plants were analyzed in our study, as well as in the studies of Popović et. al. [31] and Krstić et al. [28], there is a clear difference in the values of Cd concentrations. The reason for this could be the different localities of plant collection, their soil mineral compositions, and pH conditions, with different anthropogenic influences [43].

The average Cr content was the highest in the herbs analyzed from Kopaonik. Cr concentration varies considerably between the different herbs and the same herbs at various locations (Table 2). The minimal value of Cr was LOD (<0.08 mg/kg) for several perennial herbs from Kopaonik, and the maximum (12.1 mg/kg) was detected for lemon balm from Sokobanja, which is exceptionally high having in mind a rather low average Cr con-centration per herb at this location. The values of Cr in lemon balm from Zlatar (0.80 mg/kg), and Kopaonik (0.31 mg/kg) were also much lower compared to Sokobanja. The Tokalıoğlu [32] in Kayseri (Turkey) found a much higher amount of Cr in lemon balm than documented concentrations from Mt. Zlatar and Mt. Kopaonik but considerably lower compared to Sokobanja. The amounts of Cr in chamomile and dog rose were much higher [32] than in the same plants in the present study. In addition, a very high Cr concentration of 31 mg/kg was reported in black tea from India by de Oliveira et al. [30]. In Turkey (Kay-seri) the Cr concentrations in common nettle and St. John’s wort found by Tokalıoğlu [32] were significantly higher (8.7 and 3.70 mg/kg) than the concentration recorded in this study. The reason for the presence of a high concentration of Cr could be the result of accumulation by adsorption of Cr to iron oxides and hydroxides in soil, due to the high correlation between these metals [42]. On the other hand, the Cr amount in linden was approximately the same as in the same plant from Zlatar, but higher than in herbs from Kopaonik, and Sokobanja. It can be concluded that Cr concentrations for St. John’s wort, common nettle, and thyme differ significantly depending on the locality in Serbia [28,31]. Generally, Sharma et al. [44] reported that the concentration of Cr in roots can sometimes be 100 times higher than in the shoots, as a consequence of different mobility of Cr in the plant, which may be another reason for the presence of higher concentrations of Cr in different parts of the herbs. 

#### 3.1.2. Nickel

The average Ni content in the analyzed herbs was the highest at Zlatar and lowest at Sokobanja (Table 2). The Ni content was also higher than the content of Cd from all three locations (Table 2). A maximum of 12.2 mg/kg was observed in winter savory from Zlatar. It was confirmed that Ni concentration above 10 mg/kg in plant tissue [45] could cause different deformities in the development of plants such as slow growth, abnormalities in plant photosynthesis, and chlorosis. Foliar and soil applications of low-quality fertilizers and micronutrients are cited as the reason for the increased presence of Ni [46]. Several herbs in this study had a concentration below the LOD (<0.08 mg/kg), in particular, common nettle at all three locations. The Ni concentration in St. John’s wort found by Tokalıoğlu [32] was close to concentrations in the herbs from Zlatar and Kopaonik, and much higher than the value from Sokobanja. The value of Ni in linden was higher than the values for the same herb from all three locations, especially from Zlatar (LOD). It was also the case for chamomile, with the lowest value (LOD) at Sokobanja. The highest Ni concentration in research by Tokalıoğlu [32] was observed in common nettle, while the values for this herb in our investigation are below the LOD at all three locations. The value of Ni concentration in Turkish lemon balm was similar to, or slightly lower than, the values obtained at all three studied locations. The same was true for the dog rose. Mihaljev et al. [20] found Ni content in common nettle from Serbia of 0.738 mg/kg (leaf), and 6.034 mg/kg (root). Similar to Cd and Cr, the differences in Ni concentrations recorded by Popivić et. al. [31] and Krstić et al. [28] for St. John’s wort, common nettle, and thyme also from the territory of the Republic of Serbia, clearly indicate the dependence on the locality of the collection of herbs. The values obtained in our study for common nettle differ regarding locality: 1.0 mg/kg (Zlatar), below the LOD (<0.08 mg/kg) (Sokobanja), and 2.0 mg/kg (Kopaonik). Finally, in the present research, the highest concentration of Ni was determined in Winter savory (12.2 mg/kg; Table 2), which can be considered a potential risk to tea consumers and re-quires further analysis in order to assess the risk due to daily consumption of tea infusion.

#### 3.1.3. Lead

The average Pb content per herb follows the trend: Mt. Zlatar > Mt. Kopaonik > Sokobanja (Table 2), which corresponds to the average ^210^Pb activity radionuclide measured in the same herbs from the three locations [22,23]. The highest concentration of Pb was detected in thyme from Zlatar (49 mg/kg), and blueberry (44.5 mg/kg) from Kopaonik. The stated values exceeded the Pb limits of 3, 5, and 10 mg/kg set by the EU, European Pharmacopeia and WHO, respectively [47]. The lowest concentration (0.6 mg/kg (LOD)) was detected in St. John’s wort, linden, and comfrey from Zlatar, St. John’s wort, dog rose and juniper from Kopaonik, and chamomile, common nettle and winter savory from Sokobanja. de Oliveira et al. [30] reported the mean value of Pb in herbal teas at 2.32 mg/kg. On the other hand, the Pb concentration found by Tokalıoğlu [32] was quite lower in all herbs, and considerably lower than the corresponding Cr and Ni values, suggesting a specific soil composition, and complete absence of pollution at the location from which herbs were collected. The value of Pb in St. John’s wort was much lower than those found in our study for plants from Sokobanja, but higher than plants from Mt. Zlatar and Mt. Kopaonik (LOD). The value of Pb in linden was much lower than values in our study (which are also fairly low) obtained for herbs from Sokobanja and Kopaonik, but again higher than values from herbs from Zlatar (LOD). Similar results were found for chamomile. The value of Pb in common nettle from Turkey [32] (Tokalıoğlu, 2012) was higher than the value of the same herb from Sokobanja (LOD). The value of lemon balm from Turkey [32] was also lower than those for this herb from all three locations. The value in dog rose from Turkey [32] was similar to the same herb from Kopaonik, while values in dog rose from Sokobanja, and especially from Mt. Zlatar were quite higher. Comparably to our study, Pb concentrations recorded by Popović et al. [31] and Krstić et al. [28] for St. John’s wort, common nettle, and thyme from different local health food stores in the Republic of Serbia, did not exceed the limits of 3, 5 and 10 mg/kg set by the EU, European Pharmacopeia and WHO. The presented results once again confirm that the different localities of the collected plants, the mineralogical composition of the soil, pH values, the presence of other ions, molecules, and their mobility [46,48,49] affect the concentrations of Pb present in plants.

#### 3.1.4. Mercury

The obtained results showed that the presence of Hg was not detected in any of the samples in an amount above the detection limit of 0.06 mg/kg (Table 2). According to the literature, one of the possible explanations may be the collection time. Namely, Ordak et al. [50] found lower Hg concentrations in plants in spring (3.66–34.89 ng/g), in comparison with autumn (4.55–81.54 ng/g). Additionally, a possible explanation for the presence of Hg in plants can be soil contamination as well, which can be due to its addition across fertilizers, lime, sludges, and manures. However, the transfer of Hg from soil to plants and the dynamics of that process usually are not linear and depend on cation exchange capacity, soil pH, soil aeration, and plant species predispositions. Mercury tends to accumulate in the roots of most plants [51], but also, can accumulate in moderate amounts in the shoots [52] either due to translocation or direct absorption of the vapor form. In general, toxic heavy metal ions such as cadmium or essential metals like zinc, copper, and iron may enter the plant cells by the same process as micronutrients, competing with them for absorption. In the case of Hg, ions can enter the cells and ion channels primarily by binding to sulfur and/or nitrogen ligands [53]. However, these processes are still not well described, and data is mostly based on experiments in animal cells [9,54]. Studies of the toxicity of Hg in herbs are still scarce [55], and the cellular and molecular mechanisms that include the toxicity of Hg are practically unknown. Research is focused mainly on the primary routes of Hg exposure in humans through the consumption of food and beverages and have attracted increasing attention in recent years [27,55,56]. Especially, since it has been shown that this heavy metal can cause harmful defects in genetic material, even though low doses of Hg exposure may have adverse effects on human health [57,58].

In general, it can be concluded that the differences in the total concentration of metals among the plants primarily depend on the species and plant parts analyzed [34], but also on other conditions, such as the geological conditions, climate factors, rainfall, water, air, and soil pollution, collection time and plant age, as well as the production process of teas from plants and the method of their storage. All the mentioned reasons clearly indicate very complicated reciprocal effects, and their investigation is beyond this study.

### 3.2. Analysis and Limits of Daily Toxic Metals Intake

#### 3.2.1. Analysis and Limits of Daily Toxic Metals Intake by Consuming 200 mL Herbal Infusion

**Figure 1:**
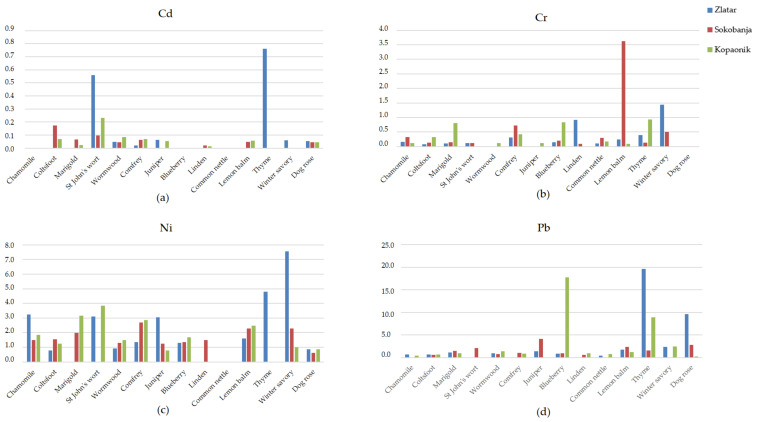
Estimated values of the daily intake (µg/day) of: (**a**) Cd, (**b**) Cr, (**c**) Ni, and (**d**) Pb due to ingestion of the 200 mL herbal infusion made from the investigated medicinal herbs.

The consumption of herbal infusions made from the investigated medicinal plants is very common in the traditional medicine of Serbia, and in many other countries. However, due to the frequent presence of increased concentrations of heavy metals in herbs, it is very important to determine the limits of daily toxic metal intake by consuming 200 mL of herbal infusion. In the present study, we have estimated the daily intake (µg/day) of Cd, Cr, Ni, and Pb due to ingestion of the 200 mL herbal infusion based on the metal solvation coefficient (Table S1). All estimated values of the daily intake of Cd, Cr, Ni, and Pb due to ingestion of the 200 mL herbal infusion made from the investigated medicinal herbs in [µg/day] are presented in Table 4 and in Figure 1. The allowed daily intake of toxic elements by humans is limited by the FAO/WHO regulations [38] (Table 5).

The presented results indicate that the daily intake of HMs in the 200 mL infusion made from the analyzed herbal samples is generally below the National Sanitation Foundation’s limit [38]. Specifically, the calculated values of the daily intake of all HMs (Table 4; Figure 1) were below the permitted intake, except Pb whose concentration was close to the limit value. The maximum calculated values were 0.762 µg/day for Cd in thyme from Zlatar, 3.630 µg/day for Cr in lemon balm from Sokobanja, 7.564 µg/day for Ni in winter savory from Zlatar, and 19.600 µg/day for Pb in thyme from Zlatar (Table 2). The herbs with a significant amount of Pb, which could produce adverse effects upon a high and/or prolonged consumption of 200 mL of infusion, are thyme from Zlatar (19.6 µg/day) and Kopaonik (8.88 µg/day), blueberry from Kopaonik (17.8 µg/day), and dog rose from Zlatar (9.6 µg/day). The presented results indicate needing to be careful about the amounts of herbal infusion made from these herbs that are drunk on a daily basis. Namely, the herbal infusion obtained from thyme from Zlatar or blueberry from Kopaonik must not be used in an amount greater than one cup of 200 mL per day. On the other hand, herbal infusion obtained from thyme from Kopaonik, or dog rose from Zlatar, must not be used in an amount greater than two cups of infusion of 200 mL per day.

#### 3.2.2. Analysis and Limits of Estimated Daily Intake (EDI) of Heavy Metals Based on the Ingestion Rate of the Herbal Infusion

Since the above results for the concentration of certain heavy metals showed the possibility of potential health risks due to the consumption of tea infusions in an amount greater than 200 mL per day, it was very important to take into account the assessment of the release of heavy metals from plants into herbal infusions. Especially, because it is known that the release of metals from the plant to water in infusions can be influenced by the quality of water (hardness) [59], temperature, pH [60], as well as the tea–water ratio. Therefore, it was very beneficial to more precisely estimate the daily intake of HMs in all investigated plants, taking into account the ingestion rate of the herbal infusion for adults (11.4 g/person/day) [6,27,29], the transfer rate of toxic metal from the medicinal herb into the infusion [6], and body weight (60 kg for adults) [6,30]. In this study, the *EDI* was calculated on the transfer of toxic metals from herbal tea into the herbal infusion and other mentioned parameters. From all three locations, the estimated daily intake is presented in Table 6. The highest *EDI* for the Cd value was estimated for St John’s wort from Kopaonik. The highest *EDI* for the Cr value was estimated for Lemon balm from Sokobanja. The highest *EDI* for Ni and Pb values were estimated for Winter savory and Thyme from Zlatar. The lowest value for Cd was from Linden (Sokobanja), for Cr was Coltsfoot (Zlatar), and for Ni and Pb was Dog rose from Sokobanja and Kopaonik, respectively.

Thus, the *EDI* values in this study were all below the oral reference dose regulated by the US EPA, suggesting that the consumption of the studied herbal infusions did not seem to pose a health risk to humans. However, by comparing the limits for toxic metal intake according to the National Sanitation Foundation draft proposal (Finished Dietary Supplement), WHO (2007) [38], the presented results suggest that more concern should be paid to the determination of the particular daily dosage infusions of HMs ingestion and their potentially negative effects. The issue of HM accumulation in the organism following long-term consumption of herbal infusions and in larger quantities is of great importance. Finally, there is no reason for concern that HMs will be accumulated in the human body, but one should be careful about long-term consumption of herbal infusions, especially in larger quantities for one day.

#### 3.2.3. Analysis of the Health Risk of the Individual Toxic Metal through Intake of Herbal Infusions

In order to assess the risk to human health from exposure to toxic metals due to herbal infusion intake, the most important thing is to calculate the hazard index. According to the calculated estimated daily intake (*EDI*), the target hazard quotient (*THQ*) was obtained for Cd, Cr, Ni, and Pb (Table 7). As shown in Table 7, the *THQ* values of individual Cd, Cr Ni, and Pb metals through the ingestion of herbal infusions were all below 1. Estimated values suggest that the daily intake of individual metals via the consumption of herbal infusions would be unlikely to have adverse health effects on people, in agreement with Zhang et al. [6].

#### 3.2.4. Analysis of Total Risk of Multiple Toxic Metals through Intake of Herbal Infusions

**Figure 2:**
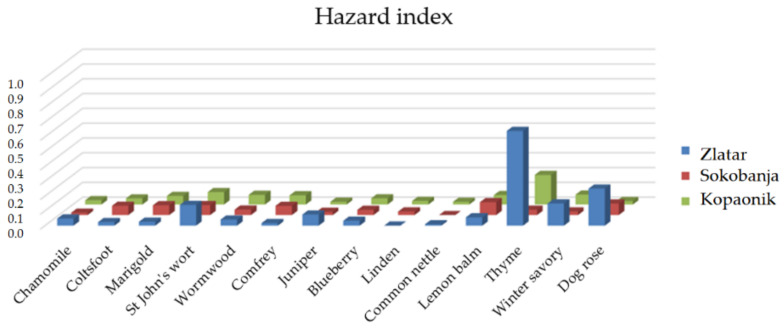
Hazard index of investigated medicinal herbs.

The results of the hazard index (*HI*) for all samples were below 1 (Table 7), so there is no health risk for taking herbal infusions from these three locations (Figure 2). The presented results do not indicate any health risk to humans when drinking the herbal infusion made from these herbs daily. Krstić et al. [28] also showed that *HI* for chamomile, St John’s wort, common nettle, thyme, winter savory, and dog rose are less than 1. *HI* presented for all teas in the paper of Zhang et al. [27] was less than 1. The intake of toxic metals through drinking herbal infusions of all medicinal herbs from Zlatar, Sokobanja, and Kopaonik is considered safe for use.

Although the *HI* values were still less than 1 and herbal infusion intake was still in the safe range, the risk of heavy metal exposure by consuming herbal infusions from certain investigated herbs still exists. The presented results suggest that more concern should be paid to determining the particular daily intake of HMs from infusion ingestion and their potentially negative effects according to the National Sanitation Foundation draft proposal (Finished Dietary Supplement) and WHO (2007) [38]. The issue of HM accumulation in the organism with long-term consumption of herbal infusions and larger quantities, is of great importance. Particular attention should be paid to the recommendations for the amount of herbal infusion consumed if the concentration of HMs is close to the limit value (for example 19,600 µg/day for Pb in thyme from Zlatar; Table 5 and Table 6). In that sense, the determination of limits for HMs in infusions made from herbal mixtures could be particularly significant to protect human health.

## 4. Conclusions

The content of heavy metals in medicinal plants is among the main criteria deter-mining their usage in traditional medicines and as herbal infusions. The concentration of five heavy metals (Cd, Cr, Ni, Hg, and Pb) in the 14 selected herbs from the three regions in central Serbia has been determined and compared with the corresponding values from the literature. The obtained results revealed that the heavy metal contents vary significantly between the investigated herbs and the locations, even for the same herbs. The largest total concentration of all five HMs per herb is found at Mt. Zlatar (the largest average concentration per herb of Pb, Ni, and Cd, and the lowest of Cr), and the lowest at Sokobanja (the lowest average concentration per herb of Pb, Ni, and Cd). Mt. Kopaonik has the largest average concentration per herb of Cr, and the lowest of Cd. The trend of the average HMs concentration per herb is Pb>Ni>Cr>Cd at all three locations.

The maximum estimated values of the daily intake of HMs in the 200 mL herbal in-fusion are 0.7616 µg/day for Cd in thyme from Zlatar, 3.63 µg/day for Cr in lemon balm from Sokobanja, 7.564 µg/day for Ni in winter savory from Zlatar, and 19.6 µg/day for Pb in thyme from Zlatar. All estimated values of the daily intake of HMs in the 200 mL infusion of the analyzed herbal samples are below the National Sanitation Foundation’s limit. In addition, the *EDI* values were all below the oral reference dose limits regulated by the US EPA, and the target hazard quotient (*THQ*) and hazard index (*HI*) for Cd, Cr Ni, and Pb were below 1. However, despite all statements, it is not recommended to drink more than one cup of the 200 mL herbal infusion made from thyme from Zlatar, and blueberry from Kopaonik, and not more than two cups of an infusion made from the other quoted herbs, due to the presence of HM in concentrations close to the limit values.

## Figures and Tables

**Table 1:** The investigated medicinal herbs with their English and Latin name, family affiliation, and the part of the herb investigated.

S. No.	Common English Name	Latin Name	Family Affiliation	Part of the Plant Investigated
1	Chamomile	*Matricaria chamomilla* L.	Asteraceae	Inflorescences
2	Coltsfoot	*Tussilago farfara* L.	Asteraceae	Leaf
3	Marigold	*Calendula officinalis* L.	Asteraceae	Inflorescences
4	St John’s wort	*Hypericum perforatum* L.	Asteraceae	Leaf and flower
5	Wormwood	*Artemisia absinthium* L.	Asteraceae	Leaf
6	Comfrey	*Symphytum officinale* L.	Boraginaceae	Root
7	Common Juniper	*Juniperus communis* L.	Cupressaceae	Fruit
8	Blueberry	*Vaccinium myrtillus* L.	Ericaceae	Leaf
9	Linden	*Tilia tomentosa* L.	Malvaceae	Inflorescences
10	Common nettle	*Urtica dioica* L.	Urticaceae	Leaf
11	Lemon balm	*Melissa officinalis* L.	Lamiaceae	Leaf
12	Thyme	*Thymus serpyllum* L.	Lamiaceae	Leaf and flower
13	Winter savory	*Satureja montana* L.	Lamiaceae	Leaf
14	Dog rose	*Rosa canina* L.	Rosaceae	Fruit

**Table 2:** The heavy metal content in the investigated medicinal herbs (mg/kg ± expanded uncertainty) from Serbia (Zlatar, Sokobanja and Kopaonik).

S. No.	Common English Name	Location	Cd	Cr	Ni	Hg	Pb
1	Chamomile	*Zlatar*	LOD	0.56 ± 0.02	5.21 ± 0.03	LOD	1.6 ± 0.1
		*Sokobanja*	LOD	1.1 ± 0.2	2.4 ± 0.5	LOD	LOD
		*Kopaonik*	LOD	0.41 ± 0.03	3.0 ± 0.1	LOD	1.0 ± 0.4
2	Coltsfoot	*Zlatar*	LOD	0.28 ± 0.04	1.27 ± 0.03	LOD	1.8 ± 0.4
		*Sokobanja*	0.62 ± 0.02	0.46 ± 0.03	2.5 ± 0.1	LOD	1.5 ± 0.2
		*Kopaonik*	0.25 ± 0.01	1.1 ± 0.1	2.0 ± 0.1	LOD	1.6 ± 0.2
3	Marigold	*Zlatar*	LOD	0.34 ± 0.01	LOD	LOD	2.9 ± 0.1
		*Sokobanja*	0.24 ± 0.03	0.50 ± 0.1	3.2 ± 0.2	LOD	3.6 ± 0.2
		*Kopaonik*	0.09 ± 0.01	2.7 ± 0.1	5.1 ± 0.2	LOD	2.3 ± 0.2
4	St John’s wort	*Zlatar*	2.00 ± 0.01	0.42 ± 0.03	5.0 ± 0.1	LOD	LOD
		*Sokobanja*	0.35 ± 0.03	0.4 ± 0.1	LOD	LOD	5.3 ± 0.2
		*Kopaonik*	0.83 ± 0.03	LOD	6.2 ± 0.1	LOD	LOD
5	Wormwood	*Zlatar*	0.17 ± 0.01	LOD	1.5 ± 0.1	LOD	2.44 ± 0.05
		*Sokobanja*	0.16 ± 0.01	LOD	2.1 ± 0.1	LOD	2.0 ± 0.2
		*Kopaonik*	0.30 ± 0.03	0.41 ± 0.02	2.4 ± 0.1	LOD	3.4 ± 0.4
6	Comfrey	*Zlatar*	0.07 ± 0.01	1.06 ± 0.03	2.2 ± 0.1	LOD	LOD
		*Sokobanja*	0.22 ± 0.02	2.4 ± 0.1	4.34 ± 0.05	LOD	2.5 ± 0.2
		*Kopaonik*	0.25 ± 0.02	1.43 ± 0.03	4.6 ± 0.1	LOD	2.1
7	Juniper	*Zlatar*	0.23 ± 0.01	LOD	4.9 ± 0.2	LOD	3.5 ± 0.1
		*Sokobanja*	LOD	LOD	2.02 ± 0.04	LOD	10.3 ± 0.4
		*Kopaonik*	0.19 ± 0.03	0.40 ± 0.1	1.26 ± 0.01	LOD	LOD
8	Blueberry	*Zlatar*	LOD	0.50 ± 0.1	2.1 ± 0.1	LOD	2.06 ± 0.1
		*Sokobanja*	LOD	0.67 ± 0.02	2.2 ± 0.1	LOD	2.3 ± 0.1
		*Kopaonik*	LOD	2.8 ± 0.1	2.7 ± 0.2	LOD	44.5 ± 2.3
9	Linden	*Zlatar*	LOD	0.65 ± 0.2	LOD	LOD	LOD
		*Sokobanja*	0.08 ± 0.01	0.3 ± 0.1	1.7 ± 0.1	LOD	1.4 ± 0.4
		*Kopaonik*	0.05 ± 0.01	LOD	LOD	LOD	2.4 ± 0.3
10	Common nettle	*Zlatar*	LOD	0.34 ± 0.03	LOD	LOD	1.0 ± 0.1
		*Sokobanja*	LOD	1.0 ± 0.03	LOD	LOD	LOD
		*Kopaonik*	LOD	0.57 ± 0.06	LOD	LOD	2.0 ± 0.1
11	Lemon balm	*Zlatar*	LOD	0.80 ± 0.1	2.6 ± 0.1	LOD	4.3 ± 0.2
		*Sokobanja*	0.17 ± 0.01	12.1 ± 0.6	3.7 ± 0.2	LOD	6.0 ± 0.4
		*Kopaonik*	0.20 ± 0.02	0.31 ± 0.03	4.0 ± 0.3	LOD	3.0 ± 0.1
12	Thyme	*Zlatar*	2.72 ± 0.03	1.32 ± 0.04	7.74 ± 0.02	LOD	49 ± 0.4
		*Sokobanja*	LOD	0.46 ± 0.03	LOD	LOD	4.0 ± 0.1
		*Kopaonik*	LOD	3.1 ± 0.1	LOD	LOD	22.2 ± 0.4
13	Winter savory	*Zlatar*	0.21 ± 0.02	4.8 ± 0.2	12.2 ± 0.6	LOD	6.0 ± 0.3
		*Sokobanja*	LOD	1.7 ± 0.3	3.7 ± 0.8	LOD	LOD
		*Kopaonik*	LOD	LOD	1.6 ± 0.1	LOD	6.2 ± 0.1
14	Dog rose	*Zlatar*	0.19 ± 0.01	LOD	1.42 ± 0.01	LOD	24 ± 0.2
		*Sokobanja*	0.16 ± 0.01	LOD	1.0 ± 0.1	LOD	7.0 ± 0.3
		*Kopaonik*	0.16 ± 0.01	LOD	1.4 ± 0.1	LOD	0.6 ± 0.2

LOD—limit of detection.

**Table 3:** The national limits of concentrations for toxic metals in herbal medicines and products (World Health Organization 2007) [38].

		Lead (Pb)	Cadmium (Cd)	Chromium (Cr)	Mercury (Hg)	Total Toxic Metals as Lead
For Herbal Medicines:						
Country	Type of product					
Canada	Herbal material [mg/kg]	10	0.3	2	0.2	/
	Finished products [mg/day]	0.02	0.006	0.02	0.02	/
China	Herbal material [mg/kg]	10	1	/	0.5	20
Malaysia	Finished herbal products [mg/kg]	10	/	/	0.5	/
Republic of Korea	Herbal material [mg/kg]	/	/	/	/	30
Singapore	Finished herbal products [mg/kg]	20	/	/	0.5	/
Thailand	Herbal material and finished herbal products [mg/kg]	10	0.3	/	/	/
WHO recommendation [mg/kg]		10	0.3	/	/	/
For other herbal products:						
National Sanitation Foundation Draft Proposal (raw dietary supplements) [mg/kg]		10	0.3	2	/	/
National Sanitation Foundation Draft Proposal (finished dietary supplements) [mg/day]		0.02	0.006	0.02	0.02	

**Table 4:** The daily intake (µg/day) of Cd, Cr, Ni and Pb due to ingestion of the 200 mL herbal infusion made from the investigated medicinal herbs.

S. No.	Common English Name	Location	Cd	Cr	Ni	Hg	Pb
			[µg/day]				
1	Chamomile	*Zlatar*	-	0.168	3.230	-	0.640
		*Sokobanja*	-	0.330	1.488	-	-
		*Kopaonik*	-	0.123	1.860	-	0.400
2	Coltsfoot	*Zlatar*	-	0.084	0.787	-	0.720
		*Sokobanja*	0.174	0.138	1.550	-	0.600
		*Kopaonik*	0.070	0.330	1.240	-	0.640
3	Marigold	*Zlatar*	-	0.102	-	-	1.160
		*Sokobanja*	0.067	0.150	1.984	-	1.440
		*Kopaonik*	0.025	0.810	3.162	-	0.920
4	St John’s wort	*Zlatar*	0.560	0.126	3.100	-	-
		*Sokobanja*	0.098	0.120	-	-	2.120
		*Kopaonik*	0.232	-	3.844	-	-
5	Wormwood	*Zlatar*	0.048	-	0.930	-	0.976
		*Sokobanja*	0.045	-	1.302	-	0.800
		*Kopaonik*	0.084	0.123	1.488	-	1.360
6	Comfrey	*Zlatar*	0.020	0.318	1.364		-
		*Sokobanja*	0.062	0.720	2.691	-	1.000
		*Kopaonik*	0.070	0.429	2.852	-	0.840
7	Juniper	*Zlatar*	0.064	-	3.038	-	1.400
		*Sokobanja*	-	-	1.252	-	4.120
		*Kopaonik*	0.053	0.120	0.781	-	-
8	Blueberry	*Zlatar*	-	0.150	1.302	-	0.824
		*Sokobanja*	-	0.201	1.364	-	0.920
		*Kopaonik*	-	0.840	1.674	-	17.80
9	Linden	*Zlatar*	-	0.195	-	-	-
		*Sokobanja*	0.022	0.090	1.054	-	0.560
		*Kopaonik*	0.014	-	-	-	0.960
10	Common nettle	*Zlatar*	-	0.102	-	-	0.400
		*Sokobanja*	-	0.300	-	-	-
		*Kopaonik*	-	0.171	-	-	0.800
11	Lemon balm	*Zlatar*	-	0.240	1.612	-	1.720
		*Sokobanja*	0.048	3.630	2.294	-	2.400
		*Kopaonik*	0.056	0.093	2.480	-	1.200
12	Thyme	*Zlatar*	0.762	0.396	4.799	-	19.60
		*Sokobanja*	-	0.138	-	-	1.600
		*Kopaonik*	-	0.930	-	-	8.880
13	Winter savory	*Zlatar*	0.059	1.44	7.564	-	2.400
		*Sokobanja*	-	0.510	2.294	-	-
		*Kopaonik*	-	-	0.992	-	2.480
14	Dog rose	*Zlatar*	0.053	-	0.880	-	9.600
		*Sokobanja*	0.045	-	0.620	-	2.800
		*Kopaonik*	0.045	-	0.868	-	0.240

**Table 5:** Limits for toxic metals intake according to National Sanitation Foundation draft proposal (Finished Dietary Supplement), WHO, 2007 [38], and the values obtained from this paper.

Heavy Metal	Limit [µg/day]	Estimated [µg/day]	Herb	Location
Cadmium (Cd)	6	0.762	Thyme	Zlatar
Chromium (Cr)	20	3.630	Lemon balm	Sokobanja
Nickel (Ni)	20	7.564	Winter savory	Zlatar
Mercury (Hg)	20	LOD	All herbs	All three
Lead (Pb)	20	19.600	Thyme	Zlatar

**Table 6:** The estimated daily intake (*EDI*) for Cd, Cr, Ni, Hg and Pb.

S. No.	Common English Name	Location	Cd	Cr	Ni	Hg	Pb
			[mg/kg/day]				
1	Chamomile	*Zlatar*	0	1.2 × 10^−5^	6.7 × 10^−4^	0	2.2 × 10^−5^
		*Sokobanja*	0	2.4 × 10^−5^	3.1 × 10^−4^	0	0
		*Kopaonik*	0	8.9 × 10^−6^	3.9 × 10^−4^	0	1.4 × 10^−5^
2	Coltsfoot	*Zlatar*	0	6.1 × 10^−6^	1.6 × 10^−4^	0	2.4 × 10^−5^
		*Sokobanja*	1.7 × 10^−5^	1.0 × 10^−5^	3.2 × 10^−4^	0	2.0 × 10^−5^
		*Kopaonik*	6.7 × 10^−6^	2.4 × 10^−5^	2.6 × 10^−4^	0	2.2 × 10^−5^
3	Marigold	*Zlatar*	0	7.4 × 10^−6^	0	0	3.9 × 10^−5^
		*Sokobanja*	6.5 × 10^−6^	1.1 × 10^−5^	4.1 × 10^−4^	0	4.8 × 10^−5^
		*Kopaonik*	2.4 × 10^−6^	5.9 × 10^−5^	6.6 × 10^−4^	0	3.1 × 10^−5^
4	St John’s wort	*Zlatar*	5.4 × 10^−5^	9.1 × 10^−6^	6.4 × 10^−4^	0	0
		*Sokobanja*	9.4 × 10^−6^	8.7 × 10^−6^	0	0	7.2 × 10^−5^
		*Kopaonik*	2.2 × 10^−5^	0	8.0 × 10^−4^	0	0
5	Wormwood	*Zlatar*	4.6 × 10^−6^	0	1.9 × 10^−4^	0	3.3 × 10^−5^
		*Sokobanja*	4.3 × 10^−6^	0	2.7 × 10^−4^	0	2.7 × 10^−5^
		*Kopaonik*	8.1 × 10^−6^	8.9 × 10^−6^	3.1 × 10^−4^	0	4.6 × 10^−5^
6	Comfrey	*Zlatar*	1.9 × 10^−6^	2.3 × 10^−5^	2.8 × 10^−4^	0	0
		*Sokobanja*	5.9 × 10^−6^	5.2 × 10^−5^	5.6 × 10^−4^	0	3.3 × 10^−5^
		*Kopaonik*	6.7 × 10^−6^	3.1 × 10^−5^	5.9 × 10^−4^	0	2.8 × 10^−5^
7	Juniper	*Zlatar*	6.2 × 10^−6^	0	6.3 × 10^−4^	0	4.7 × 10^−5^
		*Sokobanja*	0	0	2.6 × 10^−4^	0	1.4 × 10^−4^
		*Kopaonik*	5.1 × 10^−6^	8.7 × 10^−6^	1.6 × 10^−4^	0	0
8	Blueberry	*Zlatar*	0	1.1 × 10^−5^	2.7 × 10^−4^	0	2.8 × 10^−5^
		*Sokobanja*	0	1.5 × 10^−5^	2.8 × 10^−4^	0	3.1 × 10^−5^
		*Kopaonik*	0	6.1 × 10^−5^	3.5 × 10^−4^	0	6.0 × 10^−4^
9	Linden	*Zlatar*	0	1.4 × 10^−5^	0	0	0
		*Sokobanja*	2.2 × 10^−6^	6.5 × 10^−6^	2.2 × 10^−4^	0	1.4 × 10^−5^
		*Kopaonik*	1.3 × 10^−6^	0	0	0	3.2 × 10^−5^
10	Common nettle	*Zlatar*	0	7.4 × 10^−6^	0	0	1.4 × 10^−5^
		*Sokobanja*	0	2.2 × 10^−5^	0	0	0
		*Kopaonik*	0	1.2 × 10^−5^	0	0	2.7 × 10^−5^
11	Lemon balm	*Zlatar*	0	1.7 × 10^−5^	3.3 × 10^−4^	0	5.8 × 10^−5^
		*Sokobanja*	4.6 × 10^−6^	2.6 × 10^−4^	4.8 × 10^−4^	0	8.1 × 10^−5^
		*Kopaonik*	5.4 × 10^−6^	6.7 × 10^−6^	5.1 × 10^−4^	0	4.1 × 10^−5^
12	Thyme	*Zlatar*	7.3 × 10^−5^	2.9 × 10^−5^	1.0 × 10^−3^	0	6.6 × 10^−4^
		*Sokobanja*	0	1.0 × 10^−5^	0	0	5.4 × 10^−5^
		*Kopaonik*	0	6.7 × 10^−5^	0	0	3.0 × 10^−4^
13	Winter savory	*Zlatar*	5.7 × 10^−6^	1.0 × 10^−4^	1.6 × 10^−3^	0	8.1 × 10^−5^
		*Sokobanja*	0	3.7 × 10^−5^	4.8 × 10^−4^	0	0
		*Kopaonik*	0	0	2.1 × 10^−4^	0	8.4 × 10^−5^
14	Dog rose	*Zlatar*	5.1 × 10^−6^	0	1.8 × 10^−4^	0	3.4 × 10^−4^
		*Sokobanja*	4.3 × 10^−6^	0	1.3 × 10^−4^	0	9.4 × 10^−5^
		*Kopaonik*	4.3 × 10^−6^	0	1.8 × 10^−4^	0	8.1 × 10^−6^

**Table 7:** Target hazard quotient (*THQ*) and hazard index (***HI***).

S. No.	Common English Name	Location	Target Hazard Quotient (*THQ*)					Hazard Index (*HI*)
			Cd	Cr	Ni	Hg	Pb	
1	Chamomile	*Zlatar*	0	8.1 × 10^−6^	3.3 × 10^−2^	0	1.5 × 10^−2^	4.8 × 10^−2^
		*Sokobanja*	0	1.6 × 10^−5^	1.6 × 10^−2^	0	0	1.6 × 10^−2^
		*Kopaonik*	0	5.9 × 10^−6^	2.0 × 10^−2^	0	9.0 × 10^−3^	2.9 × 10^−2^
2	Coltsfoot	*Zlatar*	0	4.1 × 10^−6^	8.0 × 10^−3^	0	1.6 × 10^−2^	2.4 × 10^−2^
		*Sokobanja*	3.4 × 10^−2^	6.7 × 10^−6^	1.6 × 10^−2^	0	1.3 × 10^−2^	6.3 × 10^−2^
		*Kopaonik*	1.3 × 10^−2^	1.6 × 10^−5^	1.3 × 10^−2^	0	1.5 × 10^−2^	4.1 × 10^−2^
3	Marigold	*Zlatar*	0	4.9 × 10^−6^	0	0	2.6 × 10^−2^	2.6 × 10^−2^
		*Sokobanja*	1.3 × 10^−2^	7.3 × 10^−6^	2.1 × 10^−2^	0	3.2 × 10^−2^	6.6 × 10^−2^
		*Kopaonik*	4.9 × 10^−3^	3.9 × 10^−5^	3.3 × 10^−2^	0	2.1 × 10^−2^	5.8 × 10^−2^
4	St John’s wort	*Zlatar*	0.108	6.1 × 10^−6^	3.2 × 10^−2^	0	0	0.14
		*Sokobanja*	1.9 × 10^−2^	5.8 × 10^−6^	0	0	4.8 × 10^−2^	6.7 × 10^−2^
		*Kopaonik*	4.4 × 10^−2^	0	4.0 × 10^−2^	0	0	8.4 × 10^−2^
5	Wormwood	*Zlatar*	9.2 × 10^−3^	0	9.5 × 10^−3^	0	2.2 × 10^−2^	4.1 × 10^−2^
		*Sokobanja*	8.6 × 10^−3^	0	1.4 × 10^−2^	0	1.4 × 10^−2^	3.7 × 10^−2^
		*Kopaonik*	1.6 × 10^−2^	5.9 × 10^−6^	1.6 × 10^−2^	0	3.3 × 10^−2^	6.5 × 10^−2^
6	Comfrey	*Zlatar*	3.2 × 10^−3^	1.5 × 10^−5^	1.4 × 10^−2^	0	0	1.7 × 10^−2^
		*Sokobanja*	1.2 × 10^−2^	3.5 × 10^−5^	2.8 × 10^−2^	0	2.2 × 10^−2^	6.2 × 10^−2^
		*Kopaonik*	1.3 × 10^−2^	2.1 × 10^−5^	3.0 × 10^−2^	0	1.9 × 10^−2^	6.2 × 10^−2^
7	Juniper	*Zlatar*	1.2 × 10^−2^	0	3.2 × 10^−2^	0	3.1 × 10^−2^	7.5 × 10^−2^
		*Sokobanja*	0	0	1.3 × 10^−2^	0	9.0 × 10^−3^	2.2 × 10^−2^
		*Kopaonik*	1.0 × 10^−2^	5.8 × 10^−6^	8.0 × 10^−3^	0	0	1.8 × 10^−2^
8	Blueberry	*Zlatar*	0	7.3 × 10^−6^	1.4 × 10^−2^	0	1.9 × 10^−2^	3.3 × 10^−2^
		*Sokobanja*	0	1.0 × 10^−5^	1.4 × 10^−2^	0	2.1 × 10^−2^	3.5 × 10^−2^
		*Kopaonik*	0	4.1 × 10^−5^	1.8 × 10^−2^	0	4.0E−01	4.2 × 10^−1^
9	Linden	*Zlatar*	0	9.3 × 10^−6^	0	0	0	9.3 × 10^−6^
		*Sokobanja*	4.4 × 10^−3^	4.3 × 10^−5^	1.1 × 10^−2^	0	9.0 × 10^−3^	2.4 × 10^−2^
		*Kopaonik*	2.6 × 10^−3^	0	0	0	2.1 × 10^−2^	2.4 × 10^−2^
10	Common nettle	*Zlatar*	0	4.9 × 10^−6^	0	0	9.0 × 10^−3^	9.0 × 10^−3^
		*Sokobanja*	0	1.5 × 10^−5^	0	0	0	1.5 × 10^−5^
		*Kopaonik*	0	8.0 × 10^−6^	0	0	1.8 × 10^−2^	1.8 × 10^−2^
11	Lemon balm	*Zlatar*	0	1.1 × 10^−5^	1.7 × 10^−2^	0	3.9 × 10^−2^	5.6 × 10^−2^
		*Sokobanja*	9.2 × 10^−3^	1.7 × 10^−4^	2.4 × 10^−2^	0	5.4 × 10^−2^	8.7 × 10^−2^
		*Kopaonik*	1.1 × 10^−2^	4.5 × 10^−6^	2.6 × 10^−2^	0	2.7 × 10^−2^	6.4 × 10^−2^
12	Thyme	*Zlatar*	0.146	1.9 × 10^−5^	5.0 × 10^−2^	0	4.4 × 10^−1^	0.63
		*Sokobanja*	0	6.7 × 10^−6^	0	0	3.6 × 10^−2^	3.6 × 10^−2^
		*Kopaonik*	0	4.5 × 10^−5^	0	0	2.0 × 10^−1^	2.0 × 10^−1^
13	Winter savory	*Zlatar*	1.1 × 10^−2^	6.7 × 10^−5^	8.0 × 10^−2^	0	5.4 × 10^−2^	1.5 × 10^−1^
		*Sokobanja*	0	2.5 × 10^−5^	2.4 × 10^−2^	0	0	2.4 × 10^−2^
		*Kopaonik*	0	0	1.1 × 10^−2^	0	5.6 × 10^−2^	6.7 × 10^−2^
14	Dog rose	*Zlatar*	1.0 × 10^−2^	0	9.0 × 10^−3^	0	2.3 × 10^−1^	2.5 × 10^−1^
		*Sokobanja*	8.6 × 10^−3^	0	6.5 × 10^−3^	0	6.3 × 10^−2^	7.8 × 10^−2^
		*Kopaonik*	8.6 × 10^−3^	0	9.0 × 10^−3^	0	5.4 × 10^−3^	2.3 × 10^−2^

## Data Availability

Not applicable.

## Supplementary Materials

The following supporting information can be downloaded at: https://www.mdpi.com/article/10.3390/toxics11020198/s1, Table S1: Metal solvation coefficient Cd during the infusion preparation [6]; Table S2: Transfer rate of toxic metal from medicinal herb into the infusion [26]; Table S3: The oral reference dose regulated by the U.S. Environmental Protection Agency (US EPA) [61,62].
