# Supplementary material for: Heavy Metals Content in Selected Medicinal Plants Produced and Consumed in Serbia and Their Daily Intake in Herbal Infusions
Source: Toxics. 2023 Feb 20;11(2):198. doi: 10.3390/toxics11020198 (PMC9966102; doi:10.3390/toxics11020198)
Supplement: Supplementary file 1 [file toxics-11-00198-s001.zip › toxics-2221893-supplementary.pdf]

Supplementary materials 1

Table S1. Metal solvation coefficient C_d during the infusion preparation [6].

Heavy metals	Mass transfer in herbal infusions (%)
Cadmium (Cd)	14
Chromium (Cr)	15
Nickel (Ni)	31
Mercury (Hg)	/
Lead (Pb)	20

Table S2. Transfer rate of toxic metal from medicinal herb into the infusion [26].

Heavy metals	Transfer in herbal infusions [%]
Cadmium (Cd)	14.18
Chromium (Cr)	11.45
Nickel (Ni)	67.71
Mercury (Hg)	/
Lead (Pb)	7.11

Table S3. The oral reference dose regulated by the U.S. Enviromental Protection Agency (US EPA) [61].

Heavy metals	Reference dose [mg/kg/day]
Cadmium (Cd)	0.0005
Chromium (Cr)	1.5
Nickel (Ni)	0.02
Mercury (Hg)	/
Lead (Pb)	0.0015

The oral reference dose value for Cr was set as the value for Cr(III) due to the fact that Cr(VI) could be reduced to Cr(III) under acidic conditions in the stomach [62].
